# Management of Patients with Metastatic Bladder Cancer in the Real-World Setting from the Multidisciplinary Team: Current Opinion of the SOGUG Multidisciplinary Working Group

**DOI:** 10.3390/cancers14051130

**Published:** 2022-02-23

**Authors:** Aránzazu González-del-Alba, Antonio José Conde-Moreno, Ana M. García Vicente, Pilar González-Peramato, Estefanía Linares-Espinós, Miguel Ángel Climent, the SOGUG Multidisciplinary Working Group

**Affiliations:** 1 Department of Medical Oncology, Puerta de Hierro-Majadahonda University Hospital, Joaquin Rodrigo 1, Majadahonda, 28222 Madrid, Spain; 2 Department of Radiation Oncology, La Fe University and Polytechnic Hospital, 46026 Valencia, Spain; conde_ant@gva.es; 3 Nuclear Medicine Department, University General Hospital, 13005 Ciudad Real, Spain; angarvice@yahoo.es; 4 Department of Pathology, University Hospital La Paz, Universidad Autónoma de Madrid, 28046 Madrid, Spain; pilar.gonzalezperamato@uam.es; 5 Deparment of Urology, University Hospital La Paz, Universidad Autónoma de Madrid, 28046 Madrid, Spain; estefanialinares@gmail.com; 6 Medical Oncology Service, Fundación Instituto Valenciano de Oncología, 46009 Valencia, Spain; macliment@fivo.org

**Keywords:** metastatic bladder cancer, locally advanced bladder cancer, oligometastatic disease, immunotherapy, anti-PD-L1 antibodies

## Abstract

**Simple Summary:**

This report presents clinically relevant advances in the management of metastatic bladder cancer, which have been the focus of discussion of expert members of the Spanish Oncology Genitourinary (SOGUG) Multidisciplinary Working Group in the framework of the Genitourinary Alliance project (12GU) designed as a space for the integration of novel information in the care of bladder cancer patients. The present study is focused on different aspects regarding integration of immunotherapy especially in the patient unfit for platinum-based chemotherapy, PD-L1 assays and samples to be evaluated, role of imaging techniques in preoperative staging or re-staging, definition and treatment approach of oligometastatic disease, and rescue strategies in responders. Involvement of a dedicated multidisciplinary team in the care of patients with mBC is crucial to improve outcome.

**Abstract:**

Based on the discussion of current state of research of relevant topics of metastatic bladder cancer (mBC) among a group of experts of a Spanish Oncology Genitourinary (SOGUG) Working Group, a set of recommendations were proposed to overcome the challenges posed by the management of mBC in clinical practice. First-line options in unfit patients for cisplatin are chemotherapy with carboplatin and immunotherapy in PD-L1 positive patients. FDG-PET/CT may be a useful imaging technique in the initial staging or re-staging. In patients with oligometastatic disease, it is important to consider not only the number of metastatic lesions, but also the tumor biology and the clinical course. The combination of stereotactic body radiotherapy and immunotherapy with anti-PD-L1 monoclonal antibodies is under investigation and could improve the results of systemic treatment in patient with oligometastatic disease. Rescue treatment with curative intent could be considered in patients with oligometastatic disease after complete response on FDG-PET/CT. Metastatic disease should be evaluated using the same imaging modality over the course of the disease from diagnosis until rescue treatment. For improving the outcome of patients with mBC, the involvement of a dedicated multidisciplinary team, including urologists, pathologists, oncologists, radiologists and other specialists is of outmost importance in the daily care of these patients.

## 1. Introduction

Important advances in the understanding of the molecular mechanisms and tumor progression of urothelial carcinoma have been achieved over the past decade. Management of patients with advanced-stage, unresectable or metastatic bladder cancer (mBC) has shifted in recent years, with novel therapeutic agents available for clinical use, especially new immune checkpoint inhibitors (ICI) directed at programmed cell-death protein 1 (PD-1) or its ligand (PD-L1) with remarkable survival benefits in selected patients with metastatic disease [1]. However, a high unmet need remains for new drugs in platinum-refractory patients with advanced bladder cancer [2].

The Genito Urinary Alliance project (I2GU) was designed as a space for the integration of innovation progress in the management of patients with bladder cancer. To this purpose, each expert member of the Spanish Oncology Genitourinary (SOGUG) Multidisciplinary Working Group involved in the project reviewed the literature and redefined the state of art of his/her own area of expertise based on their clinical experience. Controversial and debatable topics of the current knowledge and approach in the care of patients with mBC were also discussed by all expert members of the SOGUG and the topics to be covered by the present review were considered. These include patients unfit for cisplatin-based chemotherapy and integration of immunotherapy, significance and role of PD-L1 assessment, treatment of oligometastatic disease, rescue therapy in respondent patients, and imaging techniques in the evaluation of response. Challenges and recommendations were reached by agreement of all participants to be applicable in clinical practice to facilitate shared decision making for individual patients with metastatic urothelial cancer.

## 2. Definition of the Patient Unfit for Platinum-Based Chemotherapy and Integration of Immunotherapy in the Management of Advanced Urothelial Cancer

The management of bladder cancer requires a multidisciplinary involvement of specialists in urology, medical oncology and radiation oncology to define the appropriate approach for individual patients based on stage and type of cancer. Patients with mBC account for 5% of newly diagnosed cases [3]. Platinum-based chemotherapy (cisplatin, carboplatin) has been for decades the treatment of choice in mBC. Approximately 40% of patients with adequate renal function are eligible for chemotherapy with cisplatin, 40% unfit for cisplatin are eligible for chemotherapy with carboplatin, and the remaining 20% are unfit for any platinum-based chemotherapy and may be treated with different options: monotherapy with paclitaxel, gemcitabine, and others [4,5,6]. In an effort to develop a consensus definition of patients with mBC unfit for cisplatin-based chemotherapy, a working group was assembled and conducted a survey of 120 international academic and community-based genitourinary oncologists [7]. Proposed eligibility criteria for mBC patients unfit for cisplatin-based chemotherapy include at least one of the following: WHO or ECOG performance status of 2 or Karnofsky status of 60–70%, creatinine clearance < 60 mL/min, Common Terminology Criteria for Adverse Events (CTCAE) v4 grade ≥ 2 audiometric hearing loss or ≥2 peripheral neuropathy, and New York Heart Association (NYHA) class III heart failure. Age is not included among the definition criteria but is an important factor to be considered in daily practice (e.g., elderly patients with comorbidities).

Renal dysfunction, poor performance status, and comorbidities may preclude frontline cisplatin-based chemotherapy in clinical practice. In the experience of a community-based cancer center of 298 patients with mBC, a first-line cisplatin-based regimen was administered to 35.9% of patients, carboplatin-based to 27.2%, non-platinum-based chemotherapy to 8.4%, and no chemotherapy in 23.8% [8]. In the IMvigor130 phase III clinical trial [9] carried out in untreated patients with mBC and randomized to atezolizumab plus platinum-based/gemcitabine chemotherapy, atezolizumab monotherapy, or placebo plus platinum-based/gemcitabine chemotherapy, 45%, 30% and 35% of patients in each group were ineligible for cisplatin-based chemotherapy, but 70%, 63% and 66% received chemotherapy with carboplatin instead of cisplatin, which may reflect real-world clinical practice.

In patients eligible for cisplatin-based chemotherapy, there are several combinations for first-line treatment, with gemcitabine/cisplatin (GC) as the most common. In a large randomized phase III study of GC versus methotrexate/vinblastine/doxorubicin/cisplatin (MVAC) [10], GC provided similar efficacy in terms of overall survival and progression-free survival compared with MVAC, but with a superior safety profile. Gemcitabine/carboplatin is also the most frequently used combination in patients unfit for cisplatin-based chemotherapy [11]. In second-line treatment, vinflunine showed a marginal efficacy as compared with best supportive care in a phase 3 clinical trial [12] and was not approved by the Food and Drug Administration (FDA) but received approval of the European Medicines Agency (EMEA). Standard therapy in mBC before the introduction of immunotherapy showed response rates of 40–50% and median survival of 12–15 months for first-line chemotherapy (GC, MVAC and paclitaxel/cisplatin/gemcitabine) in cisplatin eligible patients, 36–56% and 7–9 months for gemcitabine/carboplatin in cisplatin ineligible patients, and about 10% and 5–8 months for the single agent vinflunine in second-line therapy.

Following the requirements of the EMEA, in mBC patients ineligible for cisplatin-containing chemotherapy, PD-1/PD-L1 therapy with either atezolizumab or pembrolizumab requires the use of an FDA-approved companion diagnostic test to determine PD-L1 levels in tumor tissue. In patients with locally advanced and unresectable or mBC, two single-arm multicenter phase II studies evaluated the use of anti-PD-L1 therapy as first-line therapy in cisplatin-ineligible patients. In the IMvigor210 clinical trial of atezolizumab, at a median follow-up of 17.2 months, the objective response rate was 23% and the median overall survival was 15.9 months [13], whereas in the KEYNOTE-052 trial of pembrolizumab up to a median follow-up of 5 years, the objective response rate was 28.9% [14]. Also, in both studies durable responses were obtained. However, despite these encouraging results, response rates, progression-free survival and overall survival associated with ICIs have not been proven to be superior to carboplatin-based chemotherapy, and carboplatin-based chemotherapy remains a viable first-line treatment option in cisplatin-ineligible PD-L1-positive patients with mBC until mature data from randomized phase III of ICIs will become available [15]. Main results from first-line phase II and III trials of anti-PD-L1 agents in advanced urothelial cancer [13,16,17,18,19,20] are listed in Table 1.

In a study of maintenance treatment with the anti-PD-L1 antibody avelumab in patients who did not have disease progression with first-line chemotherapy (four to six cycles of GC or gemcitabine/carboplatin), avelumab was associated with statistically significant improvements in overall survival at 1 year as compared with best supportive care in the whole study population (hazard ratio (HR) 0.69, 95% confidence interval (CI) 0.56–0.86) and in the PD-L1-positive population (HR 0.56, 95% CI 0.40–0.79) [19].

Avelumab, durvalumab, nivolumab, atezolizumab and pembrolizumab are FDA-approved ICIs that have been evaluated as second-line options, but only atezolizumab [21] and pembrolizumab [22] in the framework of phase III randomized studies. Median overall survival was around 11 months, 20% response rate, and durable response rates at 2 years of approximately 40%.

In relation to targeted therapy, erdafitinib, an oral pan-fibroblast growth factor receptor (FGFR)-targeted agent based on relevant clinical activity in mBC patients whose tumors bear actionable FGFR alterations, data of an open-label phase 2 study in 99 patients showed confirmed response in 40% (complete response 3%, partial response 37%) [23]. The median duration of response was 5.6 months and approximately 30% of these responses were maintained for more than 12 months. At 12 months, the rate of overall survival was 55% and the rate of progression-free survival was 19%.

In the open-label phase 3 study of enfortumab vendotin (EV), an antibody-drug conjugate targeting nectin-4 was administered to patients with locally advanced or mBC with disease progression during or after treatment with PD-1/PD-L1 inhibitors and compared to chemotherapy [24]. The primary endpoint was overall survival. The median overall survival was 12.88 in the EV group vs. 8.97 months in the chemotherapy group, with a hazard ratio for death of 0.70 (95% CI 0.56–0.89; *p* = 0.001). EV significantly prolonged survival as compared with chemotherapy.

A summary of the role of immunotherapy in mBC is shown in Table 2.

### Challenges and Recommendations

- To improve definition of “unfit” considering difficulties in the integral assessment of older patients and adequate initial evaluation of the patient’s general status.
- Consolidated data are needed to determine the superiority of immunotherapy over chemotherapy for first-line treatment.
- PD-L1 expression should be measured for the selection of first-line treatment in “unfit” patients.
- In the second-line setting, immunotherapy is preferred to chemotherapy, independently of the status of PD-L1.
- The use of molecular classification based on gene expression profiles to guide therapeutic decisions in clinical practice is still limited and homogenized terminology is needed.

## 3. PD-L1 Testing in Urothelial Carcinoma

Assessment of PD-L1 levels in tumor tissue is currently recommended for a better selection of candidates for first-line treatment with the anti-PD-L1 agents atezolizumab or pembrolizumab in patients with locally advanced urothelial cancer, mBC or no candidates/refractory to cisplatin-based chemotherapy. Indication of PD-L1 testing was established based on the “one test, one drug” model, with specific quantification and interpretation criteria, and the companion diagnostic tests associated with the clinical response to the anti-PD-L1 drug. Table 3 summarizes assays for PD-L1 expression in urothelial cancer [25].

Before an PD-L1 assay, it is important to define the information that should necessary to include in the testing request form for practical, technical and cost-efficiency reasons (e.g., specification of the test, type of anti-PD-L1 drug that is intended to be prescribed, type of sample, controls). In relation to the selection of the most appropriate sample (Table 4), it is recommended the use of the most recent specimen with sufficient tumor tissue and a lower level of cauterization and necrosis. In selected cases, the use of various blocks (and even various fields) may be necessary to assess heterogeneity. There is no uniform agreement regarding the use of specimens from the primary tumor or metastatic sites, but the use of samples after neoadjuvant chemotherapy is discouraged. Positive (tonsils) and negative controls are recommended and should always be carried out according to the manufacturer’s instructions.

These assays, however, use different antibodies, immunohistochemical protocols, scoring algorithms, and cutoffs to define high/low PD-L1 expression in urothelial cancer, so that it is necessary to determine whether different therapeutic decisions may be related to the use of specific antibodies may involve different therapeutic decisions. Different studies have shown that SP263, 22C3 and 28-8 assays are analytically similar with high correlation coefficients [29,30,31,32], in contrast to the SP142 assay that shows divergent staining results, pooled percentage of agreement of 59% with SP263, 22C3 and 28-8, and fewer eligible patients identified for first-line therapy with atezolizumab [33]. Thus, patient selection for UC-1 L treatment with pembrolizumab or atezolizumab requires the use of an FDA/CE-IVD approved assay. The SP142 and 22C3 clones were approved in assays for this purpose.

The concordance of the four PD-L1 expression assays had been also evaluated in primary and metastatic bladder carcinomas. Two studies of matched pairs of transurethral resections of the bladder (TURB), cystectomy specimens and lymph node metastases showed concordant overall results, with discordance occurring more frequently after neoadjuvant therapy [34,35]. In relation to the characterization of immunohistochemical markers to recognize basal and luminal molecular subtypes, it has been shown that the basal subtype high-grade urothelial cancer has abundance of CD8+ T cells with increased expression of inhibitory markers [36]. In the pure urothelial carcinoma histology, which accounts for up to one-third of advanced cases, the three SP263, 22C3 and SP142 clones showed strong agreement in pairwise comparisons of tumor and immune cells, with high expression in urothelial carcinomas with squamous differentiation and lymphoepithelioma-like variants [37,38,39].

### Challenges and Recommendations

- Training of pathologists is indispensable to reduce intra- and inter-observer variability in PD-L1 expression assays.
- The use of specimens after neoadjuvant chemotherapy should be avoided and in case of high staining heterogeneity, examination of different fields and blocks is recommended.
- The pathological report should include integrated histological and immunohistochemical information with technical details (e.g., antibodies, platforms, cutoffs definitions) and quantitative values of the percentages of PD-L1 positivity, with a final recommendation regarding eligibility for anti-PD-L1 treatment.
- Large validation studies preferably including patients treated with ICIs are necessary to increase the use of PD-L1 assays in clinical practice.
- Assessment of molecular subtypes in addition to PD-L1 expression as well as tumor mutational load, CD8+ T cells, M2 macrophages, CTL4, and TNFβ in tumor-related fibroblasts are also promising areas for future studies.

## 4. Role of Imaging Techniques in Metastatic Urothelial Cancer

The main questions regarding the role of imaging techniques in patients with mBC refer to the indications of ^18^F-fluorodeoxyglucose positron emission tomography/computed tomography (FDG-PET/CT) in preoperative staging or re-staging (recurrence) in patients with suspicion of metastases, as well as in the assessment of response in patients with metastatic disease.

**Figure 1:**
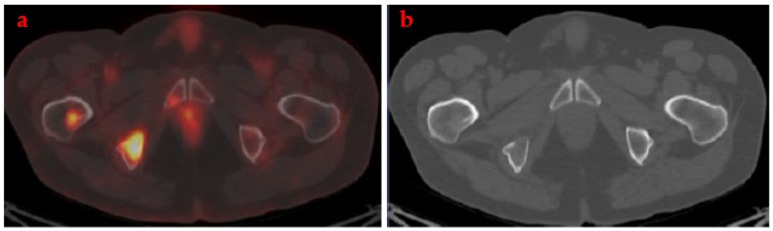
FDG-PET/CT showed metastatic bone lesions (**a**) undetected by CT (**b**).

The main advantage of FDG-PET/CT as compared with morphological images is the detection of bone metastatic disease. Although lytic bone lesions can be detected by CT, other lesions without density alterations can be evaluated by FDG uptake (Figure 1).

The usefulness of FDG-PET/CT in small lesions of <1 cm (especially in organs in motion such as the lungs) is limited, with TC or magnetic resonance imaging (MRI) providing a higher resolution, although all imaging techniques have limitations in the assessment of lesions of less than 5 mm.

The presence of lymph node involvement and distant metastasis in patients with invasive bladder carcinoma is a major determinant of survival and, therefore, a pivotal element in the therapeutic management. The rate of disseminated disease is very high increasing from 25% in T2 stage to 50% in T3 stage. Also, 50% of patients with local disease undergoing radical cystectomy will develop distant metastasis at 2 years [40]. Despite radical treatment, the 5-year overall survival of 50% probably indicates that spread of tumor cells had occurred before surgery [41].

The use of FDG-PET/CT in staging of the primary tumor currently lacks sufficient evidence for recommendation. However, the EUA-ESMO consensus statements on the management of advanced and variant bladder cancer, recommends the use of FDG-PET/CT scanning in oligometastatic disease staging when considering radical treatment [15]. FDG-PET/CT is also recommended in patients with lymph node involvement outside the pelvis or indeterminate/suspected metastatic lesions in high-risk patients [42]. In the 2020 NCCN guidelines on bladder cancer, bone scan, MRI and FDG-PET/CT is recommended to evaluate the extent of disease in symptomatic patients, at high risk of metastases or positive biomarkers of bone disease [43]. In a study of patients with advanced disease, FDG-PET/CT showed a sensitivity of 87% and specificity of 88% for the organ-based analysis and 81% and 94% for the patient-based analysis [44]. Moreover, pre- and post-PET surveys revealed that FDG-PET/CT detected more malignant disease than conventional CT/MRI in 40% of patients, and post-PET surveys showed that clinicians changed their planned management in 68% of patients based on the FDG-PET/CT results [44]. However, prospective comparative studies assessing the diagnostic reliability of the different imaging techniques for staging in advanced bladder cancer are lacking.

CT is the standard technique in the assessment of response in patients with metastatic disease, and although Response Evaluation Criteria in Solid Tumors (RECIST) has been the most widely accepted method for assessing tumor response, measurement of unidimensional diameters is a limitation of RECIST. Other limitations include changes in tumor form, assessment of unmeasurable lesions such as bone lesions, cystic transformation, heterogeneous response or no volume changes, and inability to detect tumor burden. The European Organisation for Research and Treatment of Cancer (EORTC) criteria have proven to be more sensitive in detecting complete and partial remission when compared to RECIST criteria, but hypermetabolic lesions other than tumor itself, physiological urinary FDG activity, or evaluation of organs that have high glucose utilization are limitations of EORTC [45]. On the other hand, studies assessing metabolic tumor burden are lacking.

### Challenges and Recommendations

- FDG-PET/CT is recommended in the initial staging or re-staging, in oligometastatic disease staging when considering radical treatment. FDG-PET/CT is also recommended in patients with lymph node involvement outside the pelvis or in case of indeterminate/suspected metastatic lesions in high risk patients.
- In centers in which FDG-PET/CT is not available, morphological images are also an option.
- Imaging techniques (CT, MRI, FDG-PET/CT) are complementary and it is important to select the appropriate imaging method in each case.
- The imaging technique used in the treatment follow-up of patients should be the same to that initially performed in the assessment previous to treatment.
- Studies of FDG-PET/CT using techniques for assessing metabolic tumor burden (metabolic tumor volume, glycolysis total rate) are recommended.

## 5. Treatment of Oligometastatic Disease

In 1995, Hellmann and Weichselbaum [46] proposed a clinical state of metastasis termed “oligometastases” that refers to restricted tumor metastatic capacity. According to this concept, there is an intermediate biological state of restricted metastatic capacity in which spread may be limited to specific organs and metastases may be present in limited numbers. This transitional state to dissemination may have the clinical implication that some patients affected of a significant oligometastatic state should be amenable to curative therapeutic strategies [47]. The original tumor may be controlled or uncontrolled. Based on the concept of oligometastases, the proposal of “oligo-recurrence” has a similar notion and includes the conditions of a primary site of the cancer controlled, one to several distant metastases/recurrences (usually one) in one to several organs (usually one), and one to several distant metastases/recurrences can be treated with local therapy [48]. The concept of oligo-progressive disease defines recurrence or limited progression after cytoreductive therapy or following systemic treatment, and there is relapse in a limited number of regions.

A group of international experts in diagnosis and treatment of oligometastatic disease from the EORTC and European Society for Radiotherapy and Oncology (ESTRO) OligoCare project participated in a consensus process on characterization and classification of oligometastatic disease and established a system nomenclature that cover all possible clinical situations of imaging findings with few metastases [49]. This classification based on decision tree analysis includes the three main categories of “de novo”, “repeat” and “induced” oligometastatic disease. De novo oligometastatic disease differentiates synchronous and metachronous oligo-recurrence, repeat oligometastatic disease involves response to local treatment and a small number of recurrences after a treatment-free interval, and induced oligo-progression is characterized by good responses to systemic therapy of polymetastatic disease but only a few metastases develop resistance and progress later on.

However, the optimal imaging modalities for the diagnosis and response prediction in patients with oligometastatic disease remain to be determined. Prognostic factors identified in the setting of oligometastatic disease in patients with mBC after total cystectomy include M1 disease with node-only involvement and good performance status as compared to visceral metastases (bone, lung, liver) and/or poor performance status, a solitary metastatic organ, number of metastatic lesions 3 or less, the largest diameter of metastatic foci of 5 cm or less, and no liver metastasis [50].

In relation to management of patients with oligometastatic disease, outcome is more favorable for treated than untreated patients, although no level 1 evidence is available and almost all studies are retrospective aimed to consolidate response to a previous systemic treatment or, in some cases, delay in the beginning of systemic therapy, with FDG-PET/CT as the imaging technique most used [51,52]. In a systematic review and meta-analysis to explore the role of metastasectomy in mBC based on data from 17 studies and 412 patients, metastasectomy displayed a significant better overall survival in comparison to non-surgical treatment of metastatic lesions, but only five studies were included in the meta-analysis [53]. Also, reporting of systemic treatment type, treatment schedules, and response to treatment were heterogeneous, and all except for three studies, were retrospective and non-randomized leading to a high risk of bias.

Stereotactic body radiotherapy (SBRT) associated with immunotherapy using checkpoint inhibitors (atezolizumab, avelumab, durvalumab, nivolumab, pembrolizumab) can enhanced the abscopal effect due to SBRT, and could improve the results of systemic treatment. In a randomized phase 1 trial combining pembrolizumab with either sequential or concomitant SBRT in mBC, no dose-limiting toxicity occurred and an overall response rate of 44.4% in concomitant SBRT was observed [54]. However, predicting a response is intricate with no single marker (PD-L1 or tumor burden) being sufficient to explain response or survival. Multifactorial approach combining tumor-specific and immune markers might be the key to identify who will benefit from treatment, with circulating tumor DNA (ctDNA) fraction that may serve as a surrogate for monitoring disease evolution.

### Challenges and Recommendations

- It is important to understand the concept of oligometastatic disease and to consider not only the number of metastatic lesions, but also the tumor biology and the patient’s clinical course.
- The use of ablative radiation therapy followed by systemic treatment is a recommendable strategy for the treatment of oligometastatic bladder cancer.
- Patients with M1 disease including node involvement only and good performance status have a better prognosis than patients with either visceral metastasis and/or poor perforformance status.
- Further advances in the combination of SBRT and immunotherapy with anti-PD-L1 monoclonal antibodies could improve the results of systemic treatment in patient with oligometastatic disease.

## 6. Systematization of Rescue Treatment Strategies in Responders

In general, rescue treatment is considered in patients with metastatic disease or clinically positive lymph nodes (with locally advanced disease) who had presented a clear response to systematic treatment. Rescue treatment is considered in patients with locally advanced disease or metastatic disease (synchronic or oligometastatic), which should be differentiated from recurrence appearing during the course of the disease after a previous radical treatment. Patients with recurrence are candidates to surgical rescue without systemic treatment (in case of oligometastases or metachronic metastatic disease).

There is little information in the literature regarding the role of surgery in removing metastatic lesions after response to chemotherapy. In a systematic review of 28 selected articles [55], surgery in patients with clinically positive lymph node based on data from 11 studies was associated with pooled percentages of 33%, 44% and 18% for complete clinical response, partial clinical response and pathological response, respectively. A few studies evaluated metastasectomy in lung metastasis, retroperitoneal lymph nodes and other metastatic sites, as well as cytoreductive radical cystectomy. There are important differences among studies in the percentages of partial (5–57%) and complete (24–35%) clinical response, inconsistencies in reporting pathological response, pT0 (9–30%), pN0 (37–55%), no significant differences in overall survival between cN1 and cN2-3, and with a median follow-up of 13–60 months, 5-year cancer-specific survival varies between 23% and 63%. However, as a result of a number of limitations, such as retrospective reviews of single or multiple-institution data sets, small series and span many years, unclear definition of patients who should receive cytoreductive surgery, variations in the extent of surgery, and outcomes reported for disease involvement from various regions, there are no definitive indications as to when and to who apply postchemotherapy surgery [56]. It remains challenging to provide a definitive estimate of the magnitude of this benefit from the literature, as this will vary according to the site (s) of disease and the initial tumor burden. According to NCCN guidelines [43], consolidation cystectomy or consolidation chemoradiotherapy should be offered in selected patients with complete response.

**Figure 2:**
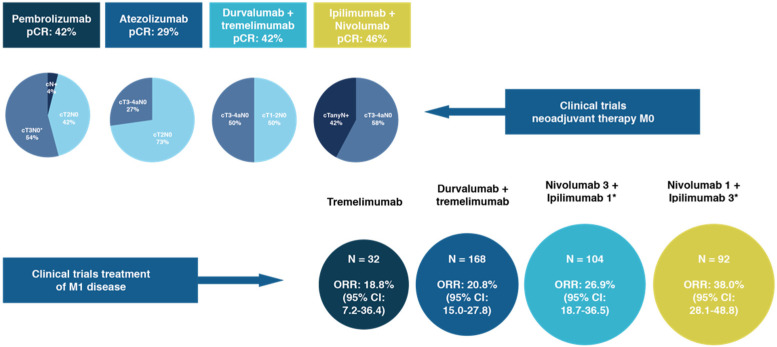
Differences in pCR in clinical trials with neoadjuvant ICIs in M0 patients and ORR in patients with M1 disease treated with ICSs alone or combined, data from [13,52,53,54,55,56,57].

Before planning surgical rescue treatment, methods for assessing clinical response is heterogeneous, with pathological complete response (pCR) used in clinical trials based on neoadjuvant therapy with ICIs [13,57,58,59] and objective response rate (ORR) in clinical trials focused on treatment of M1 patients [60,61,62] (Figure 2).

Therefore, the type of response should be defined considering different factors, such as the percentage of reduction of the initial tumor, the percentage of response of the primary tumor versus metastasis, partial response versus complete response, and type of imaging technique for assessment. In a Delphi survey study under the auspices of the EUA-ESMO Guidelines Committees [15], statements related to the role of treatment with curative intent in oligometastatic disease were discussed, with the following three proposed statements achieving consensus: (1) in a minority of patients with one metastatic lesion, cure is possible after radical treatment, (2) PET-CT scanning should be included in staging when considering radical treatment, and (3) radical treatment should be accompanied by adjuvant or neoadjuvant systemic therapy. Moreover, other statements referred to the fact that liver and bone are unfavorable oligometastatic sites for curative therapy, cure is not possible in the presence of two metastatic sites, and in metachronous oligometastatic disease, time to relapse in an important prognostic indicator.

### Challenges and Recommendations

- The benefit of consolidation surgery in overall survival and cancer-specific survival in metastatic bladder disease remains to be defined.
- Rescue treatment with curative intent could be considered in patients with oligometastatic disease after complete response on FDG-PET/CT.
- Metastatic disease should be evaluated using the same imaging modality over the course of the disease from diagnosis until rescue treatment.
- Rescue treatment includes radical cystectomy and lymphadenectomy or consolidation surgery based on radical cystectomy, lymphadenectomy and metastasectomy.
- It is necessary to establish a precise prediction of response as well as to define partial response considering volume, site, number of cycles and biomarkers.

## 7. Concluding Remarks

A tight definition of a patient unfit for cisplatin treatment remains a challenge in clinical practice. The patient’s age and different comorbidities are additional factors to be considered. In cisplatin-ineligible patients, both chemotherapy with carboplatin-gemcitabine and ICIs (atezolizumab or pembrolizumab) are valid first-line options. However, there is still no evidence of the superiority of ICIs as compared with chemotherapy.

The role of PD-L1 as a predictive biomarker of response in urothelial cancer remains unclear, but its determination is mandatory for the selection of candidates to be treated with ICIs in cisplatin-unfit patients. Training of pathologists is indispensable to reduce intra- and interobserver variations in PD-L1 expression assays. Analysis of the most recent sample with the most representative tumor material available is recommended. Although there is no agreement regarding the use of specimens from the primary tumor or metastatic sites, using samples after neoadjuvant chemotherapy is discouraged.

FDG-PET/CT is recommended in the initial staging or re-staging as well as in oligometastatic disease staging when radical treatment is considered. FDG-PET/CT is also recommended in the presence of lymph node involvement outside the pelvis or indeterminate/suspected metastatic lesions in high-risk patients. The usefulness of FDG-PET/CT in small lesions of <1 cm (especially in organs in motion such as the lungs) is limited, with TC or MRI providing a higher resolution.

In oligometastatic disease, it is important to define correctly not only the number of metastases, but also the biology and clinical course of the disease. The use of ablative radiation therapy followed by systemic treatment is a recommendable strategy for the treatment of oligometastatic bladder cancer. The combination of immunotherapy and SBRT is an investigational strategy with very promising results.

The benefit of consolidation surgery in overall survival and cancer-specific survival in metastatic bladder disease remains to be defined. Rescue treatment with curative intent could be considered in patients with oligometastatic disease after complete response on FDG-PET/CT.

Regarding the role of the hospital pharmacist, training of healthcare workers and diffusion of information are key factors for improving safety in the administration of treatment in bladder cancer patients. Chemotherapeutic agents and immunotherapy drugs are considered hazardous drugs. Appropriate measurements should be taken to minimize exposure of healthcare professionals during drug administration.

Finally, for improving the outcome of patients with mBC, the involvement of a dedicated multidisciplinary team, including urologists, pathologists, oncologists, radiologists and other specialists is of outmost importance in the daily care of these patients.

## Figures and Tables

**Table 1:** First-Line Phase II and III trials of anti-PD-L1 agents in advanced urothelial cancer.

Data		Drugs Administered							
		Atezolizumab	Pembrolizumab	Atezolizumab	Pembrolizumab	Durvalumab ± Tremelimumab		Avelumab	
First author, year, reference		Balar 2017 [13]	Vuky 2020 [16]	Galsky 2020 [17]	Powles 2021 [18]	Powles 2020 [19]		Powles 2020 [20]	
Study name		IMVIGOR210	KEYNOTE-52	IMVIGOR130	KEYNOTE-361	DANUBE		JAVELIN100	
Study type (phase)		II	II	III	III	III		III	
Treatment line		1 L cisplatin -ineligible	1 L cisplatin -ineligible	1 L both cisplatin -eligible/ineligible	1 L both cisplatin -eligible/ineligible	1 L both cisplatin -eligible/ineligible		Maintenance after 1 L both cisplatin -eligible/ineligible	
Patients (*n*)		119	374	1213	1010	1032		700	
PFSs (months)	Median	2.7	2.2	Atezo + ChT: 8.2, Pbo + ChT: 6.3, HR: 0.82	Pembro + ChT: 8.3, ChT: 7.1, HR: 0.78	ITT: D + T: 3.7, D: 2.3, ChT: 6.7	High PD-L1: D + T: 4.1, D: 2.4, ChT: 5.8	Overall population: Avelu + BSC: 3.7, BSC alone: 2.0, HR: 0.62	PD-L1 positive: Avelu + BSC: 5.7, BSC alone: 2.1, HR: 0.56
	95% CI	2.1–4.2	2.1–3.4	Atezo + ChT: 6.5–8.3 Pbo + ChT: 6.2–7.0 HR: 0.70–0.96 (*p* = 0.007)	Pembro + ChT: 7.5–8.5, ChT: 6.4–7.9, HR: 0.65–0.93 (*p* = 0.0033)	ITT: D + T: 3.4–3.8, D: 1.9–3.5, ChT: 5.7–7.3	High PD-L1: D + T: 3.6–5.7, D: 1.9–3.7, ChT: 5.6–7.2	Overall: Avelu + BSC: 3.5–5.5, BSC alone: 1.9–2.7, HR: 0.52–0.75	PD-L1 positive: Avelu + BSC: 3.7–7.4, BSC alone: 1.9–3.5, HR: 0.43–0.73
OS (months)	Median	15.9	Overall: 11.3, PD-L1 ≥ 10%: 18.5, PD-L1 < 10%: 9.7	Atezo + ChT: 16.0 Pbo + ChT: 13.4 HR 0.83	Pembro + ChT: 17.0, ChT: 14.3, HR = 0.86	ITT: D + T: 15.1, ChT: 12.1, HR: 0.85	High PD-L1: D: 14.4, ChT: 12.1, HR: 0.89	Overall: Avelu + BSC: 21.4, BSC alone: 14.3, HR: 0.69	PD-L1-positive: Avelu + BSC: NE, BSC alone: 17.1, HR: 0.56
	95% CI	10.4-NE	Overall: 9.7–13.1, PD-L1 ≥10%: 12.2–28.5, PD-L1 < 10%: 7.6–11.5	Atezo + ChT: 13.9–18.9 Pbo + ChT: 12.0–15.2 HR: 0.69–1.00 (*p* = 0.027)	Pembro+ChT: 14.5–19.5, ChT: 12.3–16.7, HR = 0.72–1.02 (*p* = 0.0407)	ITT: D + T: 13.1–18.0, ChT: 10.9–14.0, HR: 0.72–1.02 (*p* = 0·075)	High PD-L1: D: 10.4–17.3, ChT: 10.4–15.0, HR: 0.71–1.11 (*p* = 0·30)	Overall: Avelu + BSC: 18.9–26.1, BSC alone: 12.9–17.9, HR: 0.56–0.86 (*p* = 0.001)	PD-L1-positive: Avelu + BSC: 20.3-NE, BSC alone: 13.5–27.3, HR: 0.4–0.79

1 L = first-line; Atezo = atezolizumab; Avelu = avelumab; BSC = best supportive care; ChT = chemotherapy; CI = confidence interval; Cis = cisplatin; D = durvalumab; HR = hazard ratio; ITT = intention-to-treat; NE = not estimable; Pembro = pembrolizumab; OS = overall survival; Pbo = placebo; PFS = progression-free survival; T = tremelimumab.

**Table 2:** Summary of the role of anti-PD-L1 agents in advanced urothelial cancer.

- New FDA/EMA approvals: Pembrolizumab in first-line PD-L1 + cisplatin-ineligible and second-line mBC. Atezolizumab in first-line PD-L1 + cisplatin-ineligible and second-line mBC.
- Pembrolizumab is the first agent to ever show overall survival benefit in second-line therapy for mBC. - Maintenance treatment with Avelumab (anti-PD-L1) in patients who have not progressed to first-line platinum achieves improvement in overall survival.
- PD-L1 expression does not guide treatment selection in second-line treatment in urothelial cancer, because benefit with treatment has been shown in the overall population. - PD-L1 expression would “a priori” increase the likelihood of benefit from ICIs in different clinical settings, although results of studies are conflicting to validate PD-L1 as a predictor of response.
- Robust predictive biomarker is still lacking.

**Table 3:** Currently available assays for PD-L1 expression testing before treatment with anti-PD-L1 agents in patients with locally advanced or metastatic urothelial cancer.

Characteristics	Atezolizumab	Nivolumab	Pembrolizumab	Durvalumab	Avelumab
Detection antibody	SP142	28-8	22C3	SP263	SP263
IHC platform	Ventana	Dako	Dako	Ventana	Dako
Cell types scored	ICs	TCs	ICs + TCs	ICs + TCs	ICs + TCs
Cutoff definitions	PD-L1 + (ICS 2/3) ≥ 5% of ICs PD-L1+	PD-L1+ ≥ 1% TC expression	PD-L1 + CPS ≥ 10 ≥ 10 TC and IC staining	PD-L1+ ≥ 25% of ICs and TCs with membrane TC staining	PD-L1+ ≥ 5% TC staining or ≥ 10% IC staining
Estimated PD-L1 prevalence in clinical trials [20]					
Second-line	25% [21]	46% [26]	30% [22]	51% [27]	45% TC and 4.5% IC [19]
First-line	27% [13]		30% [14]	60% [28]	

IHC: immunohistochemistry; IC: immune cells; TC: tumor cells; ICS: immune cells score; CPS: combined positive score; bibliographic references in brackets.

**Table 4:** Samples to be evaluated for PD-L1 expression testing.

Samples	Antibody detected			
	SP142	22C3	28-8	SP263
Specimen	Biopsies, TRU, cystectomy	Biopsies, TRU, cystectomy	Biopsies, TRU, cystectomy	Biopsies, TRU, cystectomy
Tumor	Primary and metastasis	Primary and metastasis	Primary and metastasis	Primary and metastasis
Type of tumor	Invasive UC Papillary cancer if invasive	Invasive cancer T1-T3, high-grade papillary, CIS	Invasive UC	Invasive UC Invasive or not invasive papillary carcinoma
Sarcomatoid carcinoma	Acceptable	Acceptable		
Cytology	No	No	No	No
Decalcified bone	No	No	No	No

TRU: transurethral resection; UC: urothelial cancer; CIS: carcinoma in situ.
